# Induction of Day-Time Periodic Breathing is Associated With Augmented Reflex Response From Peripheral Chemoreceptors in Male Patients With Systolic Heart Failure

**DOI:** 10.3389/fphys.2022.912056

**Published:** 2022-05-31

**Authors:** Piotr Niewinski, Stanislaw Tubek, Bartlomiej Paleczny, Waldemar Banasiak, Piotr Ponikowski

**Affiliations:** ^1^ Institute of Heart Diseases, Wroclaw Medical University, Wroclaw, Poland; ^2^ Department of Physiology and Pathophysiology, Wroclaw Medical University, Wroclaw, Poland; ^3^ 4^th^Military Hospital, Department of Cardiology, Wroclaw, Poland

**Keywords:** peripheral chemoreceptors, periodic breathing, carotid body, induction, peripheral chemoreflex

## Abstract

Spontaneous day-time periodic breathing (sPB) constitutes a common phenomenon in systolic heart failure (HF). However, it is unclear whether PB during wakefulness could be easily induced and what are the physiological and clinical correlates of patients with HF in whom PB induction is possible. Fifty male HF patients (age 60.8 ± 9.8 years, left ventricle ejection fraction 28.0 ± 7.4%) were prospectively screened and 46 enrolled. After exclusion of patients with sPB the remaining underwent trial of PB induction using mild hypoxia (stepwise addition of nitrogen gas to breathing mixture) which resulted in identification of inducible (iPB) in 51%. All patients underwent assessment of hypoxic ventilatory response (HVR) using transient hypoxia and of hypercapnic ventilatory response (HCVR) employing Read’s rebreathing method. The induction trial did not result in any adverse events and minimal SpO_2_ during nitrogen administration was ∼85%. The iPB group (vs. non-inducible PB group, nPB) was characterized by greater HVR (0.90 ± 0.47 vs. 0.50 ± 0.26 L/min/%; *p* <0.05) but comparable HCVR (0.88 ± 0.54 vs. 0.67 ± 0.68 L/min/mmHg; *p* = NS) and by worse clinical and neurohormonal profile. Mean SpO_2_ which induced first cycle of PB was 88.9 ± 3.7%, while in sPB mean SpO_2_ preceding first spontaneous cycle of PB was 96.0 ± 2.5%. There was a reverse relationship between HVR and the relative variation of SpO_2_ during induced PB (*r* = −0.49, *p* = 0.04). In summary, PB induction is feasible and safe in HF population using simple and standardized protocol employing incremental, mild hypoxia. Pathophysiology of iPB differs from sPB, as it relies mostly on overactive peripheral chemoreceptors. At the same time enhanced HVR might play a protective role against profound hypoxia during iPB.

## Introduction

Spontaneous day-time periodic breathing (sPB) constitutes a common finding in systolic heart failure (HF). Using short-term recordings the presence of PB (defined as waxing and waning of tidal volume alternating with apnoeas and/or hypopnoeas) was documented in approximately 40–60% of HF patients (Mortara et al., 1996; La Rovere et al., 2007; La Rovere et al., 2018). Day-time PB not only affects arterial oxygen saturation (Mortara et al., 1996), but also induces marked very-low frequency oscillations in hemodynamic variables (heart rate, blood pressure) (Lorenzi-Filho et al., 1999; Leung et al., 2003). Those hemodynamic fluctuations are likely related to enhanced sympatho-respiratory coupling which is a common feature of HF ( Marcus et al., 2014 ). Finally, day-time PB has been associated with more compromised left ventricular ejection fraction (LVEF), higher levels of natriuretic peptides, poorer functional class and worse long-term prognosis (La Rovere et al., 2007; Poletti et al., 2009).

Both day-time and night-time PB share similar pathophysiology related to oversensitivity of central and peripheral chemoreceptors, changes in lung gas stores and diminished cardiac output (increased controller gain, increased plant gain, prolonged loop delay respectively) (Javaheri and Dempsey 2007; Dempsey et al., 2012). Interestingly, individuals with sPB during wakefulness are characterized by higher rate of apnoeic and desaturation events at night (La Rovere et al., 2018), which support the notion that day-time PB can be seen as a more severe phenotype within the same continuum of abnormal breathing pattern related to HF state.

It is unclear what triggers the PB pattern during wakefulness in HF patients. Possibly, supine position by reducing lung functional residual capacity leads to an increase in plant gain, thereby promoting ventilatory instability (Lillington et al., 1959; Cherniack and Longobardo 2006). On the other hand, day-time PB may be also seen in the upright position and this pattern of PB is related to the highest risk of cardiac death over long-term observation (Giannoni et al., 2020).

As day-time PB tends to be an erratic phenomenon, short-term recordings employed in the previous studies (La Rovere et al., 2007; La Rovere et al., 2018) to document its presence might underestimate the true incidence of this breathing abnormality during wakefulness. We hypothesise that in HF population among patients without apparent day-time PB in supine position, there is a subgroup of patients in whom PB might be induced and sustained by exposure to mild hypoxia. Decrease in arterial oxygen partial pressure is expected to activate peripheral chemoreceptors and therefore promote breathing instability by: 1) increasing loop gain of the system; 2) inducing hyperventilation acting as a trigger for subsequent breathing oscillations. We aim to provide physiological and clinical determinants of the inducibility of day-time PB, including hypoxic and hypercapnic ventilatory responses (HVR, HCVR respectively), and to characterize the pattern of inducible PB (iPB) in relation to sPB. This would allow for better understanding of the mechanisms leading to the different phenotypes of abnormal breathing in HF. Additionally, we intend to prove the safety and feasibility of the induction protocol devised for the study, as it could be potentially employed in the future studies targeting the novel group of patients with HF and iPB.

## Methods

### Eligibility Criteria

We prospectively enrolled HF patients meeting the following inclusion criteria: stable New York Heart Association (NYHA) functional class I-III for at least 3 months preceding the study, impaired LVEF of 15–49%, willingness and ability to complete the whole study protocol in the opinion of senior researcher. Exclusion criteria included: known sleep apnea or history of snoring/upper airways obstruction, recent (within the last month) urgent hospitalization (e.g., malignant ventricular arrhythmia, significant infection, acute coronary syndrome), severe chronic obstructive pulmonary disease, severe renal impairment, use of benzodiazepines, opioids or theophilline derivates. We studied only male subjects to avoid the possible influence of sex-related hormones on chemosensitivity (Behan et al., 2003). We excluded patients with the lowest LVEF (< 15%) due to risk of ventricular arrhythmia induction during chemosensitivity assessment. The study protocol was approved by the local Institutional Ethics Committee (Komisja Bioetyczna, Wroclaw Medical University) and conformed to the standards set by the *Declaration of Helsinki*. An informed consent has been obtained in writing from all study participants.

### Study Protocol

**FIGURE 1:**
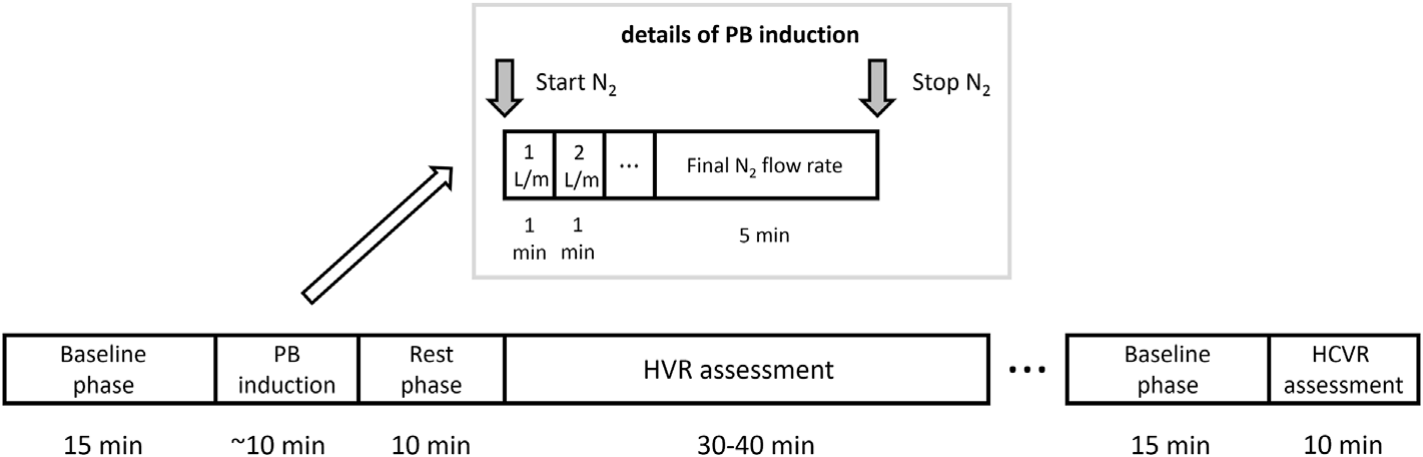
Study protocol. A graphical depiction of the timeline and details of the periodic breathing (PB) induction, hypoxic ventilatory response (HVR) and hypercapnic ventilatory response (HCVR) assessment.

The study protocol consisted of: 1) assessment of baseline hemodynamic and ventilatory parameters, 2) trial of PB induction, 3) measurement of HVR, 4) measurement of HCVR, 5) cBRS assessment, 6) laboratory tests including natriuretic peptides, 7) cardiopulmonary exercise test and 8) standard transthoracic echocardiography. Points 1-5 were performed during the same session lasting for approximately 2 h (including 15–30 min break)—see Figure 1 for details. The remaining parts of the protocol were carried out within 1 week window. Study participants underwent symptom-limited cardiopulmonary exercise test on the treadmill according to the modified Bruce protocol (Ultima, Medgraphics, St Paul, MN, United States) to determine peak oxygen consumption (peakVO_2_) and regression slope relating minute ventilation to CO_2_ output (VE/VCO_2_ slope) which was calculated from the whole period of exercise. The study (points 1-5) was performed in the morning, in the quiet, light attenuated room with stable temperature of 22°C. Participants were allowed to have a light breakfast, but asked to refrain from beverages containing caffeine for at least 12 h before the testing.

The initial part of the protocol (trial of PB induction) was unlikely to have a significant effect on the subsequent chemosensitivity assessment as: 1) it was relatively short (less than 10 min in most cases) and 2) it led to mild levels of hypoxemia which should not affect the ventilatory and hemodynamic variables for a prolonged period of time (i.e., for more than 10 min which was the length of the rest phase as per Figure 1). Moreover, before HCVR testing a longer break lasting for up to 30 min was scheduled. In cases where patient felt tired or complained of ongoing discomfort related to the use of face-mask this resting period was prolonged or in rare cases HCVR assessment was re-scheduled for the next day.

### Measurement of Hemodynamic and Ventilatory Parameters

A one-way open breathing circuit (Hans Rudolph, Inc., Shawnee, KS, United States) was used. The inhalation arm of the circuit served to administer room air or nitrogen gas (N_2_) during the PB induction trial (continuously as an addition to the breathing mixture) and for HVR assessment (transiently, pure N_2_). N_2_ administration was controlled silently using a high-pressure electric valve and a flowmeter. The exhale arm of the circuit was connected to a flow head (MLT3000L; ADInstruments, Sydney, Australia) fitted with a differential pressure transducer (FE141 Spirometer; ADInstruments) for the measurement of breathing rate (BR) and tidal volume (TV), and from this MV was calculated. HR, MBP, CO, and SVR were continuously and non-invasively recorded using a finapres technology (FMS, Enschede, Netherlands). A pulse oximeter (Radical-7; Masimo Corp., Irvine, CA, United States) with a lightweight ear clip was used to evaluate SpO_2_. Measurement of ETCO_2_ concentration was performed using a capnograph (CapStar; CWE Inc., Ardmore, PA, United States). Single-lead (lead II) electrocardiogram was obtained continuously with BioAmp device (ADInstruments). All data were collected at a sampling rate of 1 kHz (16-bit resolution) using PowerLab 16/30 (ADInstruments) and recorded on laptop computer. Hemodynamic and ventilatory parameters listed above were measured as an average from the last minute of the baseline phase as per Figure 1.

### Induction of PB

PB was defined as a presence of at least 3 consecutive cycles of hyperventilation and hypoventilation with ≥25% difference in peak and trough MV together with a typical sinusoidal pattern of ventilation (Javaheri and Dempsey 2007; La Rovere et al., 2018). This was analysed independently by two researchers and in case of doubts regarding typicality of breathing pattern was validated by the third one. In case of sustained uncertainty a subject in question was removed from the study. Subjects who presented with PB during the baseline phase (in most such cases PB was seen for more than 75% of the recording) were excluded from the induction part of the protocol and carried on with HVR assessment (sPB group).

**FIGURE 2:**
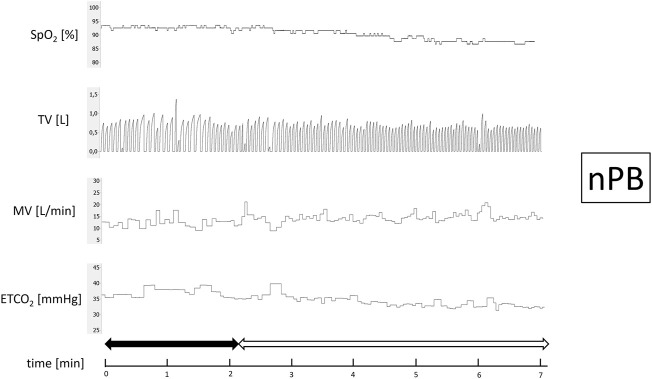
Example of non-inducible periodic breathing (nPB). Continuous recording of oxygen saturation (SpO_2_), tidal volume (TV), minute ventilation (MV), and end-tidal CO_2_ (ETCO_2_) during induction protocol. Nitrogen titration phase is depicted with solid arrow and final nitrogen flow-rate phase with blank arrow.

**FIGURE 3:**
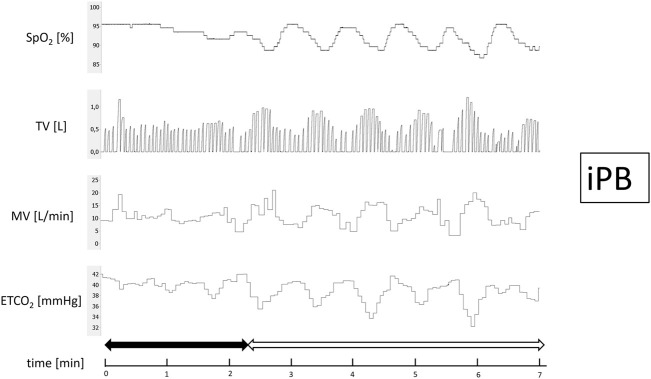
Example of inducible periodic breathing (iPB). Continuous recording of oxygen saturation (SpO_2_), tidal volume (TV), minute ventilation (MV), and end-tidal CO_2_ (ETCO_2_) during induction protocol. Nitrogen titration phase is depicted with solid arrow and final nitrogen flow-rate phase with blank arrow.

**FIGURE 4:**
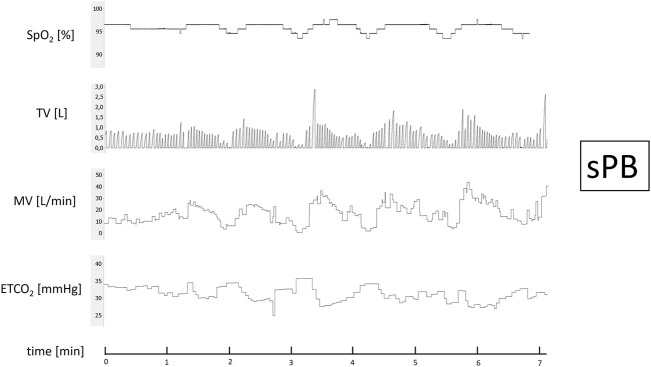
Example of spontaneous periodic breathing (sPB). Continuous recording of oxygen saturation (SpO_2_), tidal volume (TV), minute ventilation (MV), and end-tidal CO_2_ (ETCO_2_) during baseline phase recording.

**FIGURE 5:**
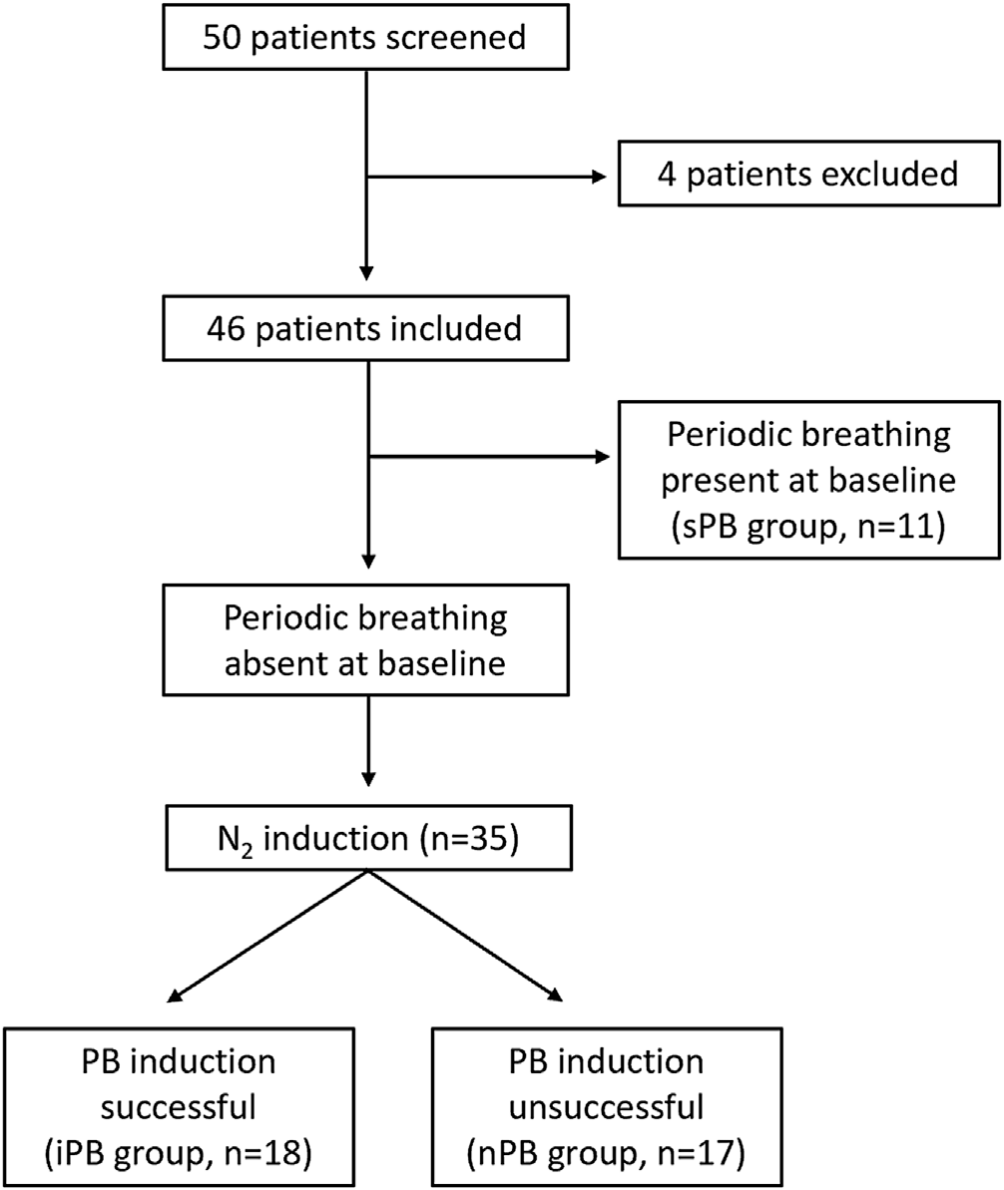
Study flowchart. Graphical presentation of the distribution of patients enrolled into the study.

After 15 min of baseline recording performed in supine position the trial of PB induction commenced. N_2_ was being added to the breathing mixture (initially consisting of room air) to gradually diminish the O_2_ concentration. The N_2_ flow was increased every minute (starting from 1 L/min in steps of 0.5–1.0 L/min) in order to achieve target SpO_2_ of 90%. From the moment when target SpO_2_ value was reached the O_2_ concentration in breathing mixture remained fixed for the next 5 min (Figure 1). The appearance of PB during this time period defined iPB group. The remaining patients in whom PB was not induced by the above protocol and was not present during baseline phase preceding induction protocol made up nPB group (see Figures 2–4 for examples and Figure 5 for patients’ distribution). We chose 90% as a target for SpO_2_ and limited the length of the induction trial to 5 min due to safety reasons as despite constant O_2_ concentration a progressive SpO_2_ decrease is usually present during prolonged hypoxic exposure (Niewinski et al., 2021). Patients were instructed to report any chest discomfort if it happened during the induction protocol. Details of the induction protocol were based on our previous work where hypoxemia of ∼90% was well tolerated over 5 min and produced PB in some of HF patients (Niewinski et al., 2021). However, in the current study for the practical reasons we used N_2_ gas tank connected to the flowmeter (instead of a gas mixer) as a source of hypoxic mixture. With this method the degree of desaturation may vary between patients despite identical N_2_ flow. Thus, N_2_ flow was titrated gradually in 0.5–1.0 L/min steps to avoid unpredictable drops in SpO_2_.

### Analysis of iPB Pattern

We reported the largest peak-trough variation in hemodynamic and ventilatory parameters within one cycle during induced PB (not necessarily the same cycle for all reported variables in a given patient). In patients with atrial fibrillation or very frequent premature beats an average from 5 s was calculated for minimal and maximal values of the hemodynamic variables within the cycle. Similarly, for MV and TV we calculated an average from 3 largest and 3 smallest breaths. The cases where complete breathing cessation took place (apnoea) in between the periods of hyperpnea were defined as Cheyne-Stokes respiration.

### Assessment of Cardiopulmonary Reflexes

#### Hypoxic Ventilatory Response

For the assessment of HVR (L/min/SpO_2_) subjects were silently switched from breathing room air to breathing pure N_2_ for 10–30 s. The length of N_2_ administrations was adjusted based on the fall in SpO_2_ caused by the first N_2_ exposure. This was repeated 5–8 times (depending on tolerability) per patient. After each N_2_ administration subjects were allowed to rest for approximately 5 min breathing room air. A single ventilatory response was averaged from the three largest consecutive breaths following the end of N_2_ administration and plotted against the associated nadir of SpO_2_ (in range between 75 and 100%) providing Point 1. Baseline values of MV and SpO_2_ were averaged from a 90 s period preceding N_2_ administration. Then, baseline MV was plotted against baseline SpO_2_ providing Point 2. The slope of the regression line linking Point 1 and Point 2 was found for each N_2_ exposure. Arithmetic average of the slopes for all N_2_ administrations was interpreted as a measure of HVR (Chua and Coats 1995).

##### Hypercapnic Ventilatory Response

**FIGURE 6:**
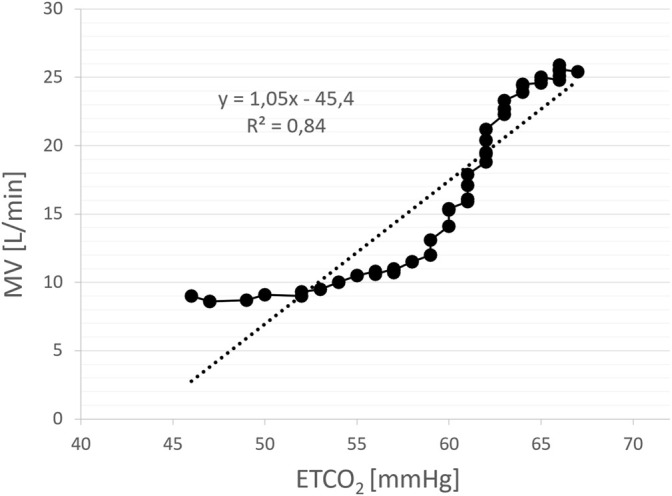
An example of the assessment of hypercapnic ventilatory response (HCVR) using rebreathing method. The slope of the regression line relating minute ventilation (MV) to end-tidal CO_2_ (ETCO_2_) constitutes a measure of HCVR (L/min/mmHg). Each solid point represents a single breath.

HCVR (L/min/mmHg) was measured using Read’s technique (Read 1967) with the subject sitting upright and rebreathing into 5-L bag filled initially with pure oxygen (to silence concomitant HVR). During the test MV and ETCO_2_ were measured simultaneously, breath-by-breath. The test ended when the patient signalled breathlessness or ETCO_2_ exceeded 70 mmHg. HCVR was calculated as a slope of the regression line relating minute ventilation to ETCO_2_ (Figure 6). The satisfactory fit of the model was confirmed with coefficient of determination (R^2^) which was 0.75 ± 0.12.

##### Cardiac Baroreflex Sensitivity

For the measurement of cBRS (ms/mmHg) continuous systolic blood pressure (SBP) and electrocardiographic (ECG) recording from the last 5 min of the initial baseline phase were used. From those all sequences of ≥3 consecutive heart beats characterized by either simultaneous increase in SBP (> 1.0 mmHg) and RR interval (*>*4.0 ms) or simultaneous decrease in SBP (> 1.0 mmHg) and RR interval (> 4.0 ms) were identified. For each sequence, a slope of the regression line relating SBP to RR interval was calculated. An average of all such slopes was considered a measure of cBRS (Davies et al., 2001). Patients with atrial fibrillation and frequent ectopic beats were excluded from the analysis.

### Statistical Analysis

Data and statistical analyses were performed with the use of Labchart Pro (AD Instruments), Matlab (Mathworks, Natick, Massachusetts) and Statistica (Statsoft, Tulsa, Oklahoma). The distribution of continuous variables was assessed using Shapiro-Wilk test. The intergroup differences were tested with the analysis of variance (ANOVA) and Kruskall-Wallis test (depending on distribution) followed by post-hoc Tukey’s HSD and Dunn’s tests, accordingly. The Jonckheere test was applied to uncover the trend within the studied groups with *a priori* ordering. The differences between dependent samples were assessed with Wilcoxon matched pairs test or t-test. Correlations were evaluated with Pearson correlation or Spearman rank correlation as appropriate. Variables are presented as means ± standard deviations. *p* value of <0.05 was considered to be statistically significant.

## Results

### Studied Patients

Fifty male patients meeting inclusion criteria were screened. Four individuals were excluded from the final analysis because of: highly irregular baseline breathing pattern (2 patients), inability to tolerate face-mask (1 patient) and an episode of atrial fibrillation during HVR testing (1 patient). The remaining patients were divided into 3 groups (non-inducible PB, inducible PB and spontaneous PB; nPB, iPB, sPB respectively) according to the criteria described above (Figure 5). Baseline data for all patients enrolled into the study (*n* = 46) are given briefly in Table 1. All patients remained on optimal pharmacological treatment (91% on ACE-I/ARB, 89% on MRA, 96% on beta-blocker) and 29% had received cardiac resynchronization therapy.

**TABLE 1:** Baseline clinical characteristics of all studied patients.

Parameter (units)	Value
Age (years)	60.8 ± 9.8
Body mass index (kg/m^2^)	28.3 ± 5.0
NYHA class I/II/III (%)	13/69/18
Ischemic etiology (%)	64
Left ventricle ejection fraction (%)	28.0 ± 7.4
NT-proBNP (pg/ml)	2723.2 ± 2329.2
peakVO_2_ (ml/kg/min)	16.5 ± 4.7

### Differences in Clinical Parameters

Patients from iPB and sPB groups were characterized by significantly lower LVEF, greater left atrial size and higher level of N-terminal pro-B-type natriuretic peptide (NT-proBNP) when compared to nPB group. Other clinical variables including: incidence of major comorbidities, pharmacological treatment and results of cardiopulmonary exercise test (including peak oxygen consumption and VE/VCO_2_ slope) did not differ between studied groups (Table 2).

**TABLE 2:** Differences in measured parameters between studied groups. *p <0.05 for inducible PB *vs.* non-inducible PB; †*p* <0.05 for spontaneous PB *vs.* non-inducible PB.

Parameter (units)	Non-inducible PB (*n* = 17)	Inducible PB (*n* = 18)	Spontaneous PB (*n* = 11)
***Clinical measures***			
Age (years)	58.4 ± 8.6	59.6 ± 11.5	66.4 ± 6.2
Body mass index (kg/m^2^)	29.8 ± 6.3	27.2 ± 4.2	27.6 ± 3.1
NYHA class I/II/III (%)	19/69/12	11/78/11	9/55/36
Atrial fibrillation (%)	38	50	36
ACE-I/ARB use (%)	81	94	100
Beta-blocker use (%)	94	100	91
MRA use (%)	88	94	82
***Laboratory tests***			
Hemoglobin (g/L)	14.1 ± 1.5	14.3 ± 1.3	14.3 ± 1.5
Creatinine (g/dl)	1.1 ± 0.2	1.0 ± 0.2	1.3 ± 0.4
Sodium (mmol/L)	140.1 ± 3.0	137.7 ± 3.8	140.4 ± 2.9
NT-proBNP (pg/ml)	1425.3 ± 1510.7	3350.4 ± 2290.2*	3670.6 ± 2726.1†
***Cardiopulmonary exercise test***			
Peak oxygen consumption (ml/kg/min)	18.8 ± 6.0	15.2 ± 3.6	14.7 ± 1.9
VE/VCO_2_ slope	41.1 ± 18.0	42.2 ± 13.1	41.1 ± 9.9
***Echocardiography***			
Left ventricle end-diastolic diameter (mm)	64.8 ± 5.4	69.5 ± 8.1	69.5 ± 9.6
Left ventricle ejection fraction (%)	33.8 ± 6.6	24.9 ± 5.7*	24.8 ± 6.0†
Left atrium diameter (mm)	46.6 ± 5.6	51.6 ± 6.3*	52.6 ± 5.0†
Severe mitral regurgitation (%)	8	6	10
***Baseline hemodynamic and ventilatory parameters***			
Cardiac output (L/min)	6.4 ± 1.0	5.6 ± 1.4	5.1 ± 1.2†
Heart rate (beats/min)	69.9 ± 11.6	71.0 ± 10.6	72.5 ± 11.9
Mean blood pressure (mmHg)	80.0 ± 9.4	82.3 ± 9.6	80.4 ± 10.2
Systemic vascular resistance (dyn·s/cm^5^)	1104.0 ± 168.7	1389.2 ± 383.5*	1428.4 ± 365.1†
End-tidal CO_2_ (mmHg)	33.3 ± 8.2	32.0 ± 5.1	30.9 ± 5.1
Oxygen saturation (%)	95.4 ± 2.7	95.9 ± 2.3	95.2 ± 2.8
Minute ventilation (L/min)	12.0 ± 6.6	11.3 ± 2.3	11.2 ± 3.9
***Cardiopulmonary reflex regulation***			
Hypoxic ventilatory response (L/min/%)	0.50 ± 0.26	0.90 ± 0.47*	0.90 ± 0.49†
Hypercapnic ventilatory response (L/min/mmHg)	0.67 ± 0.68	0.88 ± 0.54	1.16 ± 0.35†
Cardiac baroreflex sensitivity (ms/mmHg)	9.7 ± 1.9	5.0 ± 1.6*	5.1 ± 1.8†

### Differences in Baseline Hemodynamic and Ventilatory Parameters

We found higher systemic vascular resistance (SVR) in both iPB and sPB groups, however cardiac output (CO) was lower only in patients with sPB in relation to nPB group. Remaining hemodynamic and ventilatory variables were similar in all three groups (Table 2).

### Differences in Cardiopulmonary Reflex Control

**FIGURE 7:**
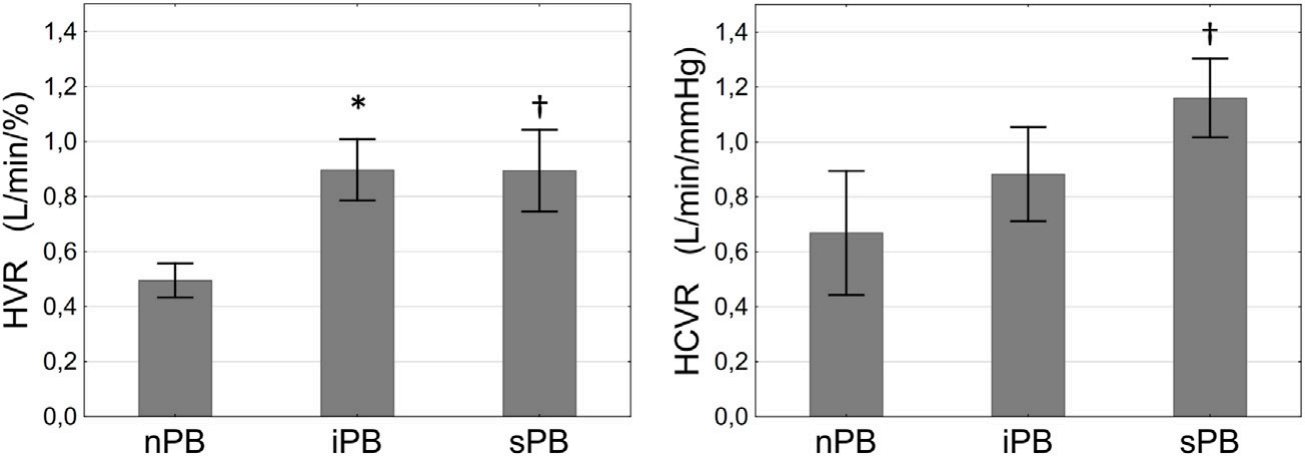
Differences in hypoxic ventilatory response (HVR, left panel) and hypercapnic ventilatory response (HCVR, right panel) between patients with non-inducible (nPB), inducible (iPB) and spontaneous periodic breathing (sPB). Data are presented as SD **±** SEM. *p <0.05 for iPB vs. nPB, †*p* < 0.05 for sPB vs. nPB.

Hypoxic ventilatory response was higher in iPB and sPB groups relative to nPB individuals. Interestingly, HCVR was greater only in sPB group but not in iPB group in relation to nPB patients. There was no difference in HVR and HCVR between sPB and iPB groups (*p* = NS for both). However, we found a significant increase in HCVR (*p* = 0.006) across the studied groups when analysed in the following order: nPB–iPB–sPB (Figure 7). Cardiac baroreflex sensitivity (cBRS) was significantly diminished in both iPB and sPB patients when compared to nPB group (Table 2).

In all studied groups the rise in MV during HCVR assessment was driven by increasing TV (0.79 ± 0.18 vs. 1.48 ± 0.34 L, *p* = 0.01; 0.90 ± 0.58 vs. 1.64 ± 0.58 L, *p* = 0.008; 0.70 ± 0.16 vs. 1.66 ± 0.53 L; *p* = 0.03 for nPB, iPB, and sPB) but not by a change in BR (15.8 ± 4.6 vs. 18.6 ± 1.8 breaths/min, *p* = 0.08; 16.7 ± 4.7 vs. 17.3 ± 5.6 breaths/min, *p* = 0.70; 18.3 ± 5.2 vs. 18.8 ± 4.9 breaths/min, *p* = 0.85 for nPB, iPB, and sPB). Analogously, hyperventilation during HVR testing was a result of rising TV (0.74 ± 0.22 vs. 1.37 ± 0.45 L, *p* <0.001; 0.80 ± 0.24 vs. 1.55 ± 0.58 L, *p* <0.001; 0.65 ± 0.13 vs. 1.32 ± 0.57 L; *p* = 0.005 for nPB, iPB, and sPB) with no significant changes regarding BR (14.8 ± 3.8 vs. 15.0 ± 3.3 breaths/min, *p* = 0.82; 15.3 ± 3.6 vs. 16.2 ± 3.6 breaths/min, *p* = 0.051; 18.4 ± 4.6 vs. 17.9 ± 4.8 breaths/min, *p* = 0.46 for nPB, iPB, and sPB).

We did not find significant differences between nPB, iPB and sPB groups in terms of HCVR test duration (288.3 ± 124.2 vs. 243.9 ± 148.3 vs. 281.3 ± 76.1 s; *p* = 0.34) and final ETCO_2_ (57.8 ± 14.3 vs. 50.3 ± 7.3 vs. 48.8 ± 5.7 mmHg; *p* = 0.37) during rebreathing.

### Inducibility of PB by Prolonged Hypoxia

**FIGURE 8:**
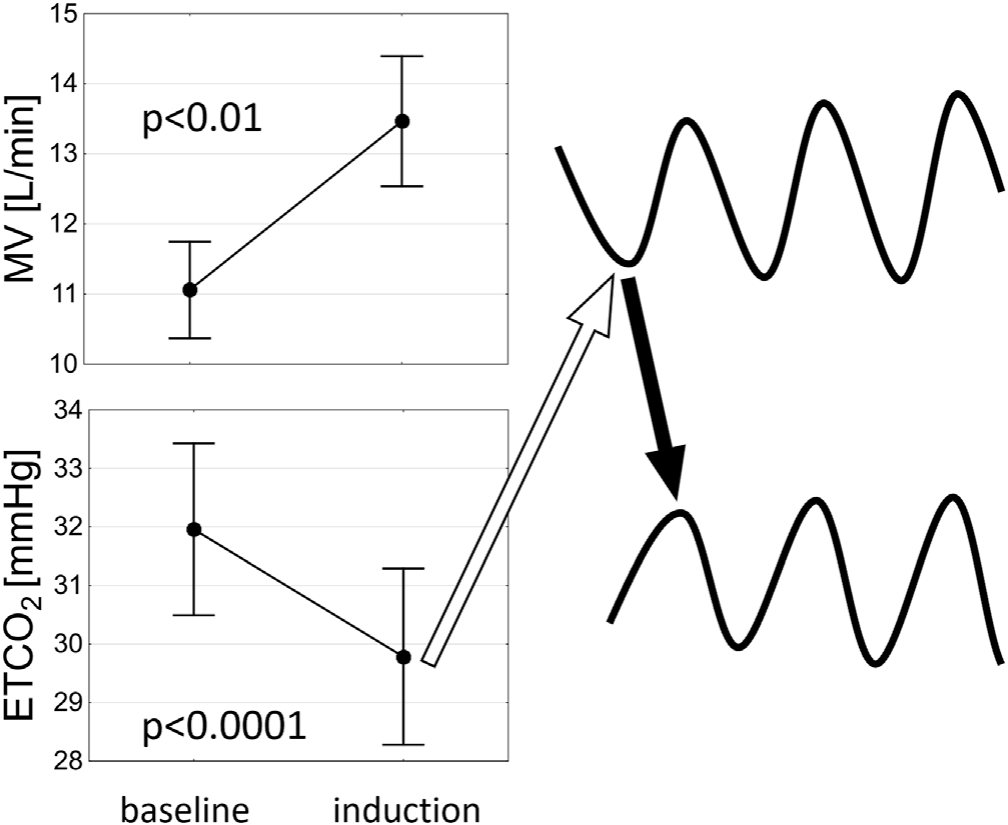
A schematic representation of the potential mechanism of PB induction. An increase in minute ventilation (MV) related to hypoxaemia leads to decrease in end-tidal CO_2_ (ETCO_2_) which results in hypoventilation (open arrow) precipitating the ongoing oscillations in ETCO_2_ and MV (solid arrow indicates a rise in ETCO_2_ following hypoventilation).

PB was induced on average at 251.9 ± 125.6 s following the initiation of the nitrogen gas (N_2_) administration. Mean blood oxygen saturation (SpO_2_) which induced first cycle of PB was 88.9 ± 3.7%. We found a significant decrease in ETCO_2_ (32.0 ± 5.7 vs. 29.8 ± 5.8 mmHg, *p* <0.0001) and an increase in MV (11.1 ± 2.7 vs. 13.5 ± 3.6 L/min, *p* <0.01) just before the first cycle of PB when compared to the beginning of induction protocol (Figure 8). Mean flow of N_2_ (added to the room air) resulting in PB induction was 3.7 ± 1.1 L/min. In sPB group mean SpO_2_ preceding first spontaneous cycle of PB was 96.0 ± 2.5%. There was no significant relationship between HVR and HCVR and the time required for PB induction (*p* = 0.70 and *p* = 0.24, respectively).

### Characteristics of the iPB Pattern

The mean relative (and absolute) variations of hemodynamic and ventilatory variables (maximal peak-trough difference) associated with iPB pattern were as follows: 28.1 ± 16.6% (1.7 ± 1.3 L/min) for CO, 31.7 ± 18.1% (412.7 ± 214.5 dyn s/cm^5^) for SVR, 14.9 ± 11.6% (11.1 ± 10.3 beats/min) for heart rate (HR), 20.6 ± 7.34% (17.2 ± 7.2 mmHg) for mean blood pressure (MBP), 18.4 ± 8.5% (5.9 ± 8.5 mmHg) for end-tidal CO_2_ (ETCO_2_), 8.5 ± 5.2% for SpO_2_ (8.1 ± 5.2%) and 94.3 ± 53.4% (11.2 ± 7.2 L/min) for minute ventilation (MV).

In 28% of cases Cheyne-Stokes pattern of respiration (presence of apnoeas) was induced.

### Safety of Induction Protocol

There was no adverse events related to PB induction using low-grade hypoxia. None of the patients reported chest discomfort. The minimal SpO_2_ during induction was 84.3 ± 5.2% in iPB and 86.4 ± 4.5% in nPB (*p* = 0.21). Time delay to SpO_2_ of 90% from the beginning of prolonged hypoxia protocol was 172.5 ± 132.8 s in iPB and 252.0 ± 208.0 s in nPB group (*p* = 0.28).

### Relationships Between Hypoxic and Hypercapnic Ventilatory Sensitivities and iPB Pattern

We found a reverse relationship between the level of HVR and the relative variation of SpO_2_ (*r* = −0.49, *p* = 0.04) and positive association between HCVR and relative variation of MBP (*r* = 0.89, *p* = 0.02) during induced PB. No other correlations were identified between HCVR, HVR and the pattern of induced PB.

## Discussion

In the current paper we present a novel phenotype of PB which could be induced by a standardized protocol employing addition of N_2_ to the breathing mixture in approximately 50% of HF patients with no apparent day-time breathing abnormalities. Patients with iPB showed distinctive clinical characteristics including poor left ventricular ejection fraction and deranged cardiorespiratory reflex control. The latter (namely exaggerated sensitivity of peripheral chemoreceptors to hypoxia i.e. HVR) may be also considered as a plausible mechanism allowing for PB induction.

As to the mechanism of PB induction by mild hypoxia, it could be speculated that hyperventilation mediated by peripheral chemoreflex (originating from sensitive to hypoxia type I cells within the carotid bodies) leads in turn to hypocapnia and thus to hypoventilation (or even to apnoea if the apnoeic threshold for CO_2_ is reached) initiating the ongoing PB in susceptible individuals such as patients with advanced HF (Figure 8). The vulnerability of HF population to the development of PB is related to alterations in various components of the feedback loop (mathematical figure describing the mechanisms of oscillations in closed-loop systems): increased controller gain [elevated chemosensitivity (Giannoni et al., 2008; Niewinski et al., 2013)], augmented plant gain [greater CO_2_ damping (Giannoni et al., 2019a) likely due to diminished lung volumes] and prolonged circulatory delay [due to low cardiac output (Naughton and Lorenzi-Filho 2009)]. The initial breathing instability related to HVR (as described above) is further fuelled by increase in the controller gain via hypoxic augmentation of the chemoreflex response to CO_2_ (Fatemian and Robbins 1998; Orr et al., 2017). This probably enables the perpetuation of ventilatory oscillations in hypoxic conditions.

The induction of PB by employing a low-level hypoxia is not completely new concept. In a study by Chadha et al. (1985) a similar protocol was employed in a small group of healthy subjects. Analogously as in our paper the addition of 2–4 L/min of N_2_ produced PB in some of the studied individuals. What differed our protocol was the use of face-mask instead of nasal cannula which allowed for the precise dosing of N_2_ and precluded varying degree of entrainment of room air through the nose as reported in cited paper. To avoid the risk of prolonged hypoxemia in HF cohort we also shortened the final phase of induction protocol to 5 instead of 10 min.

It should be also noted that despite constant content of O_2_ in the breathing mixture SpO_2_ level tends to gradually decrease over at least 10 min before reaching the plateau (Niewinski et al., 2021). Thus, it was not surprising that mean SpO_2_ which induced first cycle of PB was often lower than target level of 90% (mean = 88.9%). We believe, that such behaviour of SpO_2_ after reaching the target SpO_2_ is an inevitable part of our induction protocol.

The lack of significant relationship between HVR and HCVR and the time required to induce PB can be explained by the following factors resulting in somewhat heterogenous induction protocol: 1) starting from different baseline SpO_2_ values; 2) various responses to nitrogen addition in terms of desaturation rate (possibly related to different lung physiology between the studied patients); 3) different rates of N_2_ uptitration during induction (as per protocol steps of 0,5 or 1,0 L/min were allowed). The gradual uptitration of N_2_ amount within the breathing mixture (starting from the minuscule flow of 1 L/min for the safety reasons) also explains the prolonged time delay between the initiation of the protocol and the significant hyperventilation with subsequent decrease in ETCO_2_ required to induce the first cycle of PB (Figure 8).

We found that patients with sPB were characterized by higher HVR and HCVR when compared to nPB group. Similar finding has been already described but for PB which presence was judged based on 24 h polygraphy (Giannoni et al., 2016a). Interestingly, in the iPB group only HVR was augmented (Figure 7) implying that abnormal peripheral chemosensitivity plays a dominant role in PB induction when performed according to our protocol. It is concordant with previous studies documenting the need for the peripheral chemoreceptors in the development of apnoea following the ventilatory overshoot (Smith et al., 2003; Smith et al., 2007). The involvement of peripheral chemoreception in abnormal respiratory pattern was also reported in animal model of pacing-induced HF, where carotid body denervation markedly improved breathing stability (Marcus et al., 2014; Schultz et al., 2015). On the other hand, it could be argued that sustained hypoxia through an augmentation of HCVR (Fatemian and Robbins 1998; Orr et al., 2017) provided the lacking component of increased controller gain–namely enhanced chemosensitivity to CO_2_ during the induction trial. As hyperventilation occurring during HVR and HCVR testing was related mostly to increasing TV, it can be inferred that the differences between the studied groups in terms of ventilatory sensitivities were also predominantly related to varying responses of TV between the groups.

In contrast to iPB, in sPB group hypoxia was not necessary for the development of breathing oscillations. This is in line with the finding that HCVR was elevated in sPB but not in iPB group when compared to nPB. Therefore, it can be inferred that the mechanisms of PB is those two groups are somewhat different. The propensity for the spontaneous form of PB increases with the rising central sensitivity to CO_2_. The mild form of day-time PB (iPB group) does not require particularly high levels of HCVR but similarly as the spontaneous phenotype is characterized by clearly augmented peripheral sensitivity to hypoxia.

The highest level of HCVR noted in sPB group is consistent with the mechanism of sPB which is related to the fluctuations in arterial partial pressure of CO_2_ (getting closer and further from apnoeic threshold). Such fluctuations during day-time may be potentially triggered by hyperventilation due to supine position and concomitant pulmonary congestion–a typical feature of HF (Frasure et al., 2015). Thus, low level of HCVR could play a protective role against profound desaturations that might be seen (especially during sleep) in HF patients with co-existing PB. This is further supported by an interesting study by Giannoni et al. where administration of centrally acting agent buspirone (5-HT_1A_ receptor agonist) led to 41% reduction in CO_2_ chemosensitivity and to a significant reduction in oxygen desaturation index during daytime and nighttime (Giannoni et al., 2021). Furthermore, the trend for decreasing HCVR across sPB > iPB > nPB groups (as shown in our study) could explain the relatively small level of desaturation during induction protocol (mean SpO_2_ variation of 8%) and the rather low incidence rate for the induction of Cheyne-Stokes breathing pattern (28%).

Clinical profile of patients with iPB showed number of similarities to sPB group. Firstly, both iPB and sPB patients presented with comparable systolic function of the left ventricle which was significantly lower than in nPB. Secondly, the level of neurohormonal derangement reflected by NT-proBNP level was also similar and >2-fold higher comparing to nPB group. Analogously, cBRS did not differ between iPB and sPB, but was twice smaller than in nPB. The latter finding is not surprising as the reverse relation between HVR and baroreflex sensitivity has been reported before (Ponikowski et al., 2001). Finally, both groups experiencing PB showed similar hemodynamic and ventilatory parameters including higher (than in nPB) level of systemic vascular resistance–most likely mirroring enhanced sympathetic activity in patients with abnormal breathing pattern (Joho et al., 2012). Interestingly, the untoward effect of PB on hemodynamic variables is not necessarily related to repeated hypoxemia but rather to respiratory modulation of the autonomic tone (sympatho-respiratory coupling), as administration of O_2_ sufficient to eliminate hypoxic dips has no effect on HR or BP (Leung et al., 2006). While there was no difference in CO between iPB and nPB patients, the sPB group presented with significantly lower CO when compared to nPB. Prolonged circulatory delay related to low CO together with additional input from CO_2_ sensitive chemoreceptors could explain the propensity of sPB patients for the maintenance of breathing oscillations during day-time (Orr et al., 2017). Based on the above observations regarding clinical phenotype it could be speculated that the presence of iPB might be related to worse long-term prognosis similarly as in sPB (Poletti et al., 2009). This however remains to be confirmed in further studies.

The standardized protocol used for PB induction proved to be safe and feasible. First cycle of PB occurred on average after ∼4 min from the start of N_2_ administration and the whole procedure took less than 10 min in most of the cases. PB induction was well tolerated with no adverse events noted during that phase. We did observe one episode of atrial arrhythmia, but that took place during HVR testing. Another patient was not able to tolerate the face mask used for the measure of MV–this however was revealed even before PB induction started. Stepwise addition of N_2_ to the breathing mixture led to minimal SpO_2_ of ∼85% which can be considered safe from the clinical point of view, especially when experienced for a period of few minutes. Comparable levels of SpO_2_ may be encountered for example during long-haul commercial air flights (Hobkirk et al., 2013).

Analysis of the pattern of induced PB brought to light few intriguing observations. We found that while maximal variation in SpO_2_ was modest (8.5%) the concomitant maximal variations in hemodynamic variables such as CO and SVR were quite striking (∼30%). Similar degree of variability in the echocardiographic measures of left ventricular function and pulmonary artery pressure during PB has been reported before (Giannoni et al., 2019b). The pronounced oscillations in hemodynamic variables are possibly a reflection of augmented sympatho-respiratory coupling. Such phenomenon was elegantly described in HF model where it was clearly related to the enhanced sensitivity of peripheral chemoreflex ( Marcus et al., 2014 ). This was confirmed following the ablation of carotid bodies which significantly reduced the degree of the coupling between ventilation and sympathetic nerve activity. Thus, peripheral chemoreceptors may be seen as a crucial link between oscillatory changes in ventilation, sympathetic tone and hemodynamic parameters.

We reported an unintuitive finding of reverse relationship (*r* = −0.49) between the level of HVR and the maximal variation of SpO_2_ during induced PB. It is likely that increased HVR (through ventilatory augmentation) might play a protective role against profound desaturations occurring at the time of PB. Therefore, augmented HVR on the one hand predisposes to PB but on the other hand prevents from repeated hypoxaemic insults. Additionally, a positive relation between HCVR and maximal amplitude in MBP was noted. As individuals with greater HCVR present with higher sympathetic tone (Meguro et al., 2007), it could be hypothesized that adrenergically mediated inotropic and vasoconstrictor response to hypercapnia are together responsible for the greater blood pressure variability during PB. The exaggerated MBP response to oscillatory changes in ventilation could be also related to enhanced sympatho-respiratory coupling ( Leung et al., 2003 )—this however was not directly assessed in our study.

Our findings might have several potential clinical applications. Firstly, identification of iPB strongly suggests performing polysomnography testing to fully assess the degree of breathing abnormalities, which are likely to be present due to similar clinical profile to the patients with sPB. Secondly, patients with iPB are probably prone to development of an anomalous breathing pattern, when exposed to prolonged hypoxia e.g., at high altitude or during long-haul flights. Thirdly, one could speculate that iPB can be considered as a state preceding more advanced forms of PB and as such requires a close follow-up.

Presented study is not without limitations. We did not perform sleep polygraphic studies which would definitely provide much deeper insight into the advancement and pathophysiology of PB in enrolled individuals. Instead, we focused only on the day-time PB which by itself has been showed to carry important prognostic significance (Poletti et al., 2009). Analysis of the components of the feedback loop was limited to the controller gain assessment (HCVR and HVR measurements). Possibly, an assessment of plant gain and circulatory delay (Giannoni et al., 2019b) would be advantageous in unravelling the mechanisms behind iPB. Similarly, the measurement of HCVR at hypoxic conditions (not performed in our study) would add to the understanding of hypoxic PB induction. Our study was focused only on HF population in which revealing a novel phenotype of PB might have clinical and possibly even prognostic meaning. Inclusion of healthy volunteers would definitely shed more light on the complex mechanisms of PB induction. Also, we did not evaluate the effect of induction stimulus in sPB group which could provide an additional insight into the mechanism of PB. Finally, our study lacked female participants which could have influenced our results as gender-related differences have been reported as to the prevalence of day-time PB in HF population ( Giannoni et al., 2020 ).

To summarize, we showed that iPB is a common phenomenon in HF patients, where it can be revealed using simple, quick and safe protocol employing N_2_ administration. The clinical picture of patients with iPB resembles individuals with the spontaneous form of day-time PB. However, the pathophysiology of iPB and sPB is different. While iPB relies mostly on augmented ventilatory response to hypoxia arising from peripheral chemoreceptors, sBP also requires enhanced (in comparison to HF patients without any form of PB) hypercapnic reactivity provided by oversensitive central chemoreceptors. The latter tended to gradually rise across the spectrum of breathing abnormalities described in our study.

## Data Availability

The raw data supporting the conclusions of this article will be made available by the authors, without undue reservation.
